# Individual and unit level insights from hospital staff ratings on structural empowerment, leadership-management performance, well-being, and quality of care

**DOI:** 10.1186/s12913-024-11945-6

**Published:** 2024-11-28

**Authors:** Karin Lundin, Maria Engström, Bernice Skytt, Annika Strömberg, Marit Silén

**Affiliations:** 1 Faculty of Health and Occupational Studies, University of Gävle, Gävle, Sweden; 2 Medicine College, Lishui University, No. 1 Xueyuan Road, Lishui City, China; 3 Department of Public Health and Caring Sciences, Uppsala University, Uppsala, Sweden; 4 Faculty of Education and Business Studies, University of Gävle, Gävle, Sweden

**Keywords:** Hospital, Leadership, Nurses, Quality of care, Structural conditions, Well-being, Nursing

## Abstract

**Background:**

Leadership and access to structural empowerment are known to influence the work life experiences of staff and quality of care. Knowledge about relationships between specific factors of structural empowerment, leadership and management, staff well-being and quality of care at both an individual and unit level is scarce.

**Aim:**

To study the relationship between staff-rated access to empowering structures, leadership and management performance, well-being, and quality of care in hospital settings measured at the individual level and aggregated at the unit level.

**Methods:**

A cross-sectional correlative design was applied. Questionnaire data from 331 randomized hospital nursing staff working at 38 units in 25 hospitals in Sweden were analyzed using bivariate correlations and general estimation equation (GEE) models.

**Results:**

Results from the bivariate analysis of relationships confirmed earlier research. In the GEE models, some unexpected results were found and differences between the individual and unit levels. Adding management and leadership as independent factors in the second model showed few relationships of significance to the outcome variables.

**Conclusion:**

Results confirm the importance of staff access to empowering structures in relation to well-being and quality of care. Differences and similarities were shown when studying these relationships at both the individual and unit level. The findings feature implications for hospital management to promote staff access to empowering structures. The findings provide information on how these structures relate to the individual and the unit; information that could be useful when planning or implementing strategies with the aim to promote staff well-being and care quality. The non-significant results for leadership and management in relation to staff outcomes in the GEE-models, raise questions for further research where a shift from individual to organizational focused performances within the field of leadership is implied.

## Background

With a global shortage within the nursing workforce [1], and reports of a strained work situation in hospitals worldwide [2], there is a need for a further understanding of nursing staffs’ work life experiences to enable improved working conditions, staff well-being, and the quality of care. The first-line managers (FLMs) are in a key position to influence the work life experiences of nursing staff through their management and leadership [3–5]. With their ability to give staff access to necessary prerequisites to perform their work [6, 7], they are also in a key position to improve the quality of the care [8, 9]. The present study focuses on and investigates staff-rated access to empowering structures, leadership and management performance, staff well-being, and the quality of the care in hospital settings at individual and unit level.

Earlier studies showing the relationships between structural empowerment and the well-being of nursing staff originated mainly in North America [10], and the foundation was laid by Laschinger and colleagues [11–14]. However, more recent studies can be found from both western and eastern societies [15–17]. They all point to the importance of giving nursing staff access to empowering structures, which according to Kanter’s organizational theory [18] are e.g., opportunities (to gain new skills and knowledge), information (related to one’s work and organization), support (feedback and help from superiors and colleagues), resources (time, materials and personnel), formal power (e.g., a visible work role of importance) and informal power (e.g., a network of alliances within and outside the workplace. According to the theory of structural empowerment, staff who have access to empowering structures feel empowered and this kind of power is essential for their ability to get their work done, and thereby also reach the goals of the organization [18]. Laschinger and colleagues have previously shown how nursing staffs’ access to structural empowerment and perceived leadership at the unit level can influence staff well-being outcomes at the unit and the individual level [19]. In one of their studies, Laschinger et al. [14] found how job satisfaction among individual nurses was predicted by both individual and contextual factors when studying structural empowerment relationships with data from unit and individual levels. In the present study, the concept of well-being includes the presence of both positive conditions with perceived job satisfaction [20] and thriving at work [21, 22], and a negative condition with perceived stress symptoms [23]. Positive and negative conditions have been used to represent outcomes of staff well-being in previous studies. Positive significant relationships between structural empowerment and job satisfaction at the individual level has been shown in several hospital nursing staff studies [24–26]. The study by Dahinten et al. [25] that examined workplace empowerment in relation to job satisfaction among nursing staff, found that access to empowering structures was shown to be the strongest independent predictor for individual job satisfaction among nurses followed by the empowering behaviors of their leaders. In the model of thriving at work by Spreitzer et al. [22], they describe how certain enabling conditions in the workplace should be present for the individual to thrive e.g., information sharing, decision-making discretion, knowledge, feedback etc., which are conditions that are in line with the empowering structures described by Kanter [18] and Laschinger et al. [13]. Furthermore, findings from a meta-analysis that studied a variety of contexts has shown that, an antecedent to thriving at work is support from colleagues and managers [27]. In regard to negative influence on well-being, i.e., stress symptoms in the present study, previous studies have shown statistically significant negative relationships between access to empowering structures and individual levels of stress and burnout among nurses [15–17]. In the study by Orgambídez and Almeida [15], it was found that hospital nursing staffs’ access to informal power had a direct relationship to burnout and an indirect relationship through access to opportunities and resources. Previous studies have also shown the importance of structural empowerment in relation to the quality of the care perceived by both staff [14, 28–30] and patients [8]. Quality of care could be referred to the degree to which healthcare services meet established standards and effectively address the needs of patients, or as outlined by Donabedian [31], encompassing the components of *structure* as physical and organizational characteristics of the healthcare system, the *processes* involved in delivering care, and the *outcomes* achieved for patients. In the present study, quality of care is studied according to the individualized care scale (ICS) validated by Suhonen et al. [32], which could be seen as reflecting the component of *process*, showing whether staff perceive that care has been delivered in a proper way. Previous research has shown how nursing staff that asses structures of their professional practice environment, with similar features to the structures described in Kanter’s theory, as more positive also report to deliver a more individualized care to their patients [33].

Research in nursing has also presented evidence regarding the influence of leadership on the quality of care [34] and the well-being of nursing staff [3]. Statistically significant relationships between leadership and the quality of care assessed by both the nursing staff [29] and the patients [35] has been found in previous research. Different leadership styles have shown different relationships regarding staff outcomes of well-being and the quality of care. Positively significant relationships have been shown with leadership styles characterized as being relational oriented [3]. Negatively significant relationships have been found when hospital nursing staff have reported the leadership behavior of their managers to be, as defined by Labrague, toxic [36, 37]. Furthermore, studies have shown how the leadership of hospital FLMs, both direct and indirect through their influence on staff access to empowering structures, is related to nursing staff job satisfaction at an individual level [38, 39]. Additional important features presented in the thriving at work model [21] have shown to be useful for managers when they work to enable well-being among the nursing staff [40]. It has been found that the leadership of FLMs can enhance the ability of hospital nursing staff at an individual level to thrive at work [41]. Demographic factors and work attributes that have previously been reported to influence nursing staff well-being and the quality of care, and are included in the analysis of the present study, are: age [23, 27], gender [42], years of experience from work in the profession [43] and years of experience at the current hospital unit [44].

To summarize, it has been shown that in regard to staff well-being and the quality of care, it is important that nursing staff have access to empowering structures, and FLMs execute leadership characterized as being relational-building. However, although the existing research has studied these concepts and their relationships separately and combined, designs where regression models are constructed with the factors of structural empowerment and various measures of leadership performance as independent variables are still of interest to explore. Which factors of structural empowerment and how these factors could contribute to achieve staff well-being and quality of care is still unclear. Moreover, previous studies often present the results of the outcomes at an individual level. To translate this knowledge to unit-level practice, there is a need for further research at different levels of data where especially the unit level should be addressed. Studies examining these relationships with aggregated data at the unit level for both the independent and outcome variables is scarce. Such knowledge is important when designing workplace interventions that have the aim to develop healthy working conditions for the nursing staff and the quality of care delivered to the patients at hospitals. Since a more comprehensive exploration of the factors that contribute to nursing staff well-being and the quality of care is still required, this study has approached self-rated nursing staff well-being and quality of care as outcomes related to a combination of organizational and social factors (empowering structures, management, and leadership). Thus, the aim was to study relationships between staff-rated access to empowering structures, leadership and management performance, well-being and quality of care in hospital settings measured at the individual level and aggregated at the unit level.

## Methods

### Design

A cross-sectional and correlative design was applied. Guidelines from Strengthening the Reporting of Observational Studies in Epidemiology (STROBE) Statement for observational studies were followed [45].

### Sample and settings

The sample was derived from a list of all of the medical and surgical hospital units, both private and public, in Sweden (*n* = 752). From the 752 units, 150 units were randomized for inclusion. Approval for participation was requested from the heads of the departments from the randomized units, and 56 department heads gave their written permission. Together, these department heads were in charge of 78 FLMs. From those, 38 FLMs agreed to participate. Fifteen staff members from each of those units, which resulted in a total of 570 eligible staff members, were randomly selected and asked to participate in the survey. This sample size was considered to ensure representativeness and to yield 80% statistical power at a 95% confidence interval. Inclusion criteria for the participants were employment of at least 50% on the dayshift or a combined day and night shift of at least 3 months at the current hospital unit. The 38 included hospital units consisted of surgical units *n* = 15 and medical units *n* = 23. The units were located in 25 different hospitals that were spread throughout Sweden.

### Data collection procedure

The FLMs at the participating units chose when the questionnaires were to be sent out (September to November 2016). The FLMs gave oral information about the study to their staff at workplace meetings. A letter giving information about the study, a questionnaire and a stamped return envelope was sent by mail to the workplace mailbox of the 15 randomized staff members. Questionnaires were coded. Non-responders were sent two reminders with a two week interval. A questionnaire that began with questions regarding participant characteristics was followed by the instruments.

### Instruments

A Swedish version [30] of The Conditions for Work Effectiveness Questionnaire (CWEQ-II) [13], was used to measure structural empowerment. The questionnaire consists of 19 items and 6 factors (opportunity, information, support, resources, formal power, and informal power). Items were scored using a 5-point Likert scale with 1 (none) to 5 (a lot). Scores from the six subscales were averaged and thereafter summed to obtain the total empowerment score, where higher scores represent a more desirable state. In previous research, construct validity has been found to be satisfactory and Cronbach’s alpha values for the total scale has been reported to exceed 0.70 [30]. In the present study, the Cronbach’s alpha values for the total scale was 0.91 and for the subscales: opportunity 0.75, information 0.90, support 0.86, resources 0.82, formal power 0.76 and informal power 0.74.

The Leadership and Management Inventory (LaMI) [46] was used to measure staff estimates of their FLMs’ leadership and management performance. LaMI consists of 25 items and two factors: management and leadership. Responses are rated on a 5-point scale (1-5). All factors and total scale are scored to values between 20 and 100 (summarizing the item scores in a factor, dividing by the maximum and multiplying by 100), where higher scores represent a more desirable state. The scale has been tested for validity and reliability with satisfactory results [46]. In the present study, the Cronbach’s alpha values for the total scale was 0.96, for the factor management 0.93 and for leadership 0.97.

To measure staff-rated quality of care, the individualized care scale – nurse (ICS-nurse) instrument [32, 43] was used. The ICS-nurse is a bipartite questionnaire with 34 items equally divided in two dimensions (ICS-A-nurse 17 items and ICS-B-nurse 17 items). ICS-A-nurse explores nurses’ views on how nurses support patient individuality through nursing activities in general. ICS-B-nurse explores the extent to which nurses perceive the care they have provided to patients during their most recent working shift as individual care. The ICS-A-nurse and the ICS-B-nurse have 5-point Likert type scales that range from 1 (strongly disagree) to 5 (strongly agree). Both scales consist of three sub-scales: (1) clinical situation (seven items); (2) personal life situation (four items); and (3) decisional control over care (six items). Scores from each dimension are summed to obtain a total score of the two dimensions separately. Higher scores represent a more individualized care to the patients. Previous research has shown acceptable validity and reliability results with the Cronbach’s alpha coefficients ≤ 0.70 [32]. In the present study Cronbach’s alpha values for ICS-A was 0.90 and for ICS-B 0.91.

For the measurement of work-related well-being, instruments covering stress symptoms, job satisfaction and thriving at work were used. To measure stress symptoms, one factor from the Satisfaction with Work Questionnaires was used [23]. All items are rated on a 5-point Likert scale that range from 1 (very often) to 5 (never). Total scores of the items are summarized, divided by the highest possible score in the factor and multiplied by 100. Higher scores being representative of a more desirable state. The scale has shown evidence of validity and reliability with Cronbach’s alpha values over 0.70 [23]. In the present study, the Cronbach’s alpha value was 0.66.

Job satisfaction was assessed using the Brief Index of Affective Job Satisfaction (BIAJS) questionnaire [20], which is a four-item measure of affective job satisfaction. All items are rated on a five-point scale ranging from 1 (strongly disagree) to 5 (strongly agree). The item scores are then averaged to receive a single overall job satisfaction score, where higher scores represent a more desirable state. Two distracter items, removed from analyses, are used to help attenuate common method variance. Previous tests of validity and reliability have shown acceptable results with the Cronbach’s alpha values exceeding 0.70 [20]. In the present study, the Cronbach’s alpha value was 0.74.

To measure thriving at work, a scale developed by Porath et al. [21] was used. The scale has 10 items, half of which represent the learning factor, and the other half represent the vitality factor. The response alternatives range from 1 (totally disagree) to 7 (totally agree). The mean values of the item scores in each factor and total score for the instrument are calculated, with higher scores indicating a greater degree of thriving at work. Support for construct validity has been reported in previous research [21]. The Cronbach’s alpha values for the total scale has been reported to exceed 0.70 [21, 47]. In present study, the Cronbach’s alpha value for the total scale was 0.90.

### Data analysis

Descriptive statistics were used to describe the participants’ demographics (Table 1). Bivariate correlations were measured using Spearman’s Rho (Tables 2 and 3). Given the hierarchical structure of data, two sets of general estimating equation (GEE) models were used to measure multivariate relationships, and accounting for potential clustering [48]. Potential clustering accounted for in the models were hospital and working unit. In the first set of models, individual level measures of all included variables were used (Tables 4 and 5). In the second set, unit-aggregated data for the same variables were used (Tables 6 and 7), i.e., each individual received the mean value of their unit. Independent variables in the GEE models were the factors from structural empowerment (Model 1), and the factors leadership and management (added in Model 2). The outcome variables were stress symptoms, job satisfaction, thriving at work and quality of care ICS-A and ICS-B. Variables measuring demographic variables were included as covariates in the GEE models if they had a *p*-value < 0.1 in the bivariate analyses with the outcomes (Model 3). The significance level was set to p-value < 0.05 in the GEE-models. To check for the potential risk of common method variance, Harman’s single-factor test was done where all items from each of this study’s included constructs were loaded into an exploratory factor analysis [49]. No general factor emerged as being accountable for the majority of the covariance between the measures, and the loadings of the first factor ranged between 28.8 and 37.4%, which is an indication of a low risk for response biases [49]. Visual inspection of the histograms and scatterplots of the residuals revealed no major deviances from a normal distribution. All values of the variance inflation factor (VIF) in the multivariate analyses were below 3.6, which indicated no problems with multicollinearity [50]. Missing values were handled by replacing them with the group mean, and cases with missing values > 50% in a factor or instrument were excluded (*n* = 19) from the analyses. All statistical analyses were performed using IBM SPSS Statistics version 27 (IBM Corp, Armonk, New York).

## Results

A total of 312 staff members from 38 units (2–14 employees under each first line manager) participated in the study (response rate 55%). Most of the participants were female (93.2%) and worked as registered nurses (57.6%), while 41.8% worked as nurse assistants. The participants total mean age was 42 years with a range of 21–70 years. The years of experience of work in their current profession ranged from 3 months to 46 years (mean 16.3 years). One unit was excluded when analyzing unit aggregated data, since only one participant responded. This left 37 units with mean values from a total of 311 staff members. For participants characteristics see Table 1.

**Table 1:** Characteristics of the participants

**Age**, years			
Mean (SD), minimum–maximum	41.8	(12.6)	21 years to 70 years
**Sex**, *n *(%)			
Female	290	(93.2)	
Male	21	(6.8)	
**Professionals**, *n* (%)			
Registered Nurse	179	(57.6)	
Assistant Nurse	130	(41.8)	
**Level of Education**, *n* (%)			
Specialist nurse education	16	(5.1)	
Bachelor’s degree	66	(21.2)	
Master’s degree	4	(1.3)	
**Work experience**, years Mean (SD), minimum–maximum			
Years worked in profession	16.3	(13.7)	3.5 months to 46 years
Years worked at unit	8.9	(9.6)	3 months to 52.5 years
Years worked under current first-line manager	3.8	(3.8)	3 months to 20 years

When the sum does not add up to 312, there are internal missing data

The bivariate correlation analyses revealed expected relationships for all independent and dependent variables with the use of both individual (Spearman’s Rho from 0.13 to 0.50) and unit-aggregated data (0.13 to 0.82), except for the variables; leadership and quality of care A (individual data), information and quality of care A and B (B only individual level data), and opportunities and stress symptoms (both individual and unit-aggregated data). Furthermore, for the unit-aggregated data, non-significant results were found for; management and quality of care A and B, and resources and quality of care A (Tables 2 and 3). For relationships between demographic variables and the outcomes see Tables 2 and 3.

**Table 2:** Relationships among study variables, Spearman’s rho (individual level)

Variables	Stress symptoms		Job satisfaction		Thriving at work		Quality of care (Individualized care scale-A)		Quality of care (Individualized care scale-B)	
Opportunities	0.068	0.219	**0.353**	**< 0.001**	**0.472**	**< 0.001**	**0.180**	**0.001**	**0.176**	**0.002**
Information	**0.244**	**< 0.001**	**0.166**	**0.003**	**0.212**	**< 0.001**	0.095	0.089	0.103	0.066
Support	**0.338**	**< 0.001**	**0.447**	**< 0.001**	**0.499**	**< 0.001**	**0.271**	**< 0.001**	**0.185**	**< 0.001**
Resources	**0.433**	**< 0.001**	**0.341**	**< 0.001**	**0.331**	**< 0.001**	**0.137**	**0.014**	**0.131**	**0.019**
Formal power	**0.284**	**< 0.001**	**0.422**	**< 0.001**	**0.434**	**< 0.001**	**0.213**	**< 0.001**	**0.194**	**< 0.001**
Informal power	**0.224**	**< 0.001**	**0.328**	**< 0.001**	**0.470**	**< 0.001**	**0.272**	**< 0.001**	**0.295**	**< 0.001**
Management	**0.336**	**< 0.001**	**0.301**	**< 0.001**	**0.284**	**< 0.001**	**0.167**	**0.003**	**0.193**	**< 0.001**
Leadership	**0.270**	**< 0.001**	**0.341**	**< 0.001**	**0.337**	**< 0.001**	0.084	0.135	**0.139**	**0.014**
Age	**0.193**	**< 0.001**	**0.121**	**0.029**	−0.049	0.381	0.034	0.547	−0.047	0.403
Years in profession	**0.208**	**< 0.001**	0.084	0.135	−0.045	0.428	0.093	0.097	−0.025	0.660
Years at unit	0.057	0.317	−0.042	0.459	**−0.128**	**0.024**	0.053	0.350	−0.103	0.070

Statistically significant values are marked with bold text

**Table 3:** Relationships among study variables, Spearman’s rho (unit level)

Variables	Stress symptoms		Job satisfaction		Thriving at work		Quality of care (Individualized care scale-A)		Quality of care (Individualized care scale-B)	
Opportunities	0.093	0.102	**0.484**	**< 0.001**	**0.604**	**< 0.001**	**0.287**	**< 0.001**	**0.460**	**< 0.001**
Information	**0.348**	**< 0.001**	**0.148**	**0.009**	**0.130**	**0.022**	0.024	0.668	**0.204**	**< 0.001**
Support	**0.511**	**< 0.001**	**0.436**	**< 0.001**	**0.474**	**< 0.001**	**0.252**	**< 0.001**	**0.249**	**< 0.001**
Resources	**0.819**	**< 0.001**	**0.509**	**< 0.001**	**0.371**	**< 0.001**	0.054	0.347	**0.249**	**< 0.001**
Formal power	**0.567**	**< 0.001**	**0.516**	**< 0.001**	**0.478**	**< 0.001**	**0.145**	**0.010**	**0.404**	**< 0.001**
Informal power	**0.289**	**< 0.001**	**0.367**	**< 0.001**	**0.449**	**< 0.001**	**0.203**	**< 0.001**	**0.482**	**< 0.001**
Management	**0.538**	**< 0.001**	**0.437**	**< 0.001**	**0.306**	**< 0.001**	−0.089	0.119	0.038	0.507
Leadership	**0.515**	**< 0.001**	**0.373**	**< 0.001**	**0.231**	**< 0.001**	**−0.176**	**0.002**	0.018	0.758
Age	0.035	0.536	−0.044	0.440	−0.082	0.148	0.017	0.769	0.032	0.577
Years in profession	0.036	0.533	−0.051	0.378	−0.091	0.113	0.049	0.394	0.036	0.537
Years at unit	−0.041	0.484	−0.048	0.411	**−0.112**	**0.052**	**0.108**	**0.062**	0.054	0.347

Statistically significant values are marked with bold text

## Individual level

The first model, Model 1, showed relationships between the factors of structural empowerment and staff well-being (Table 4) and quality of care (Table 5). The factors opportunities, support and resources, were all statistically significantly related to all three outcomes of well-being (Table 4) and the factors opportunities, support, and informal power were statistically significantly related to quality of care. For the factor opportunities, the relationships were all positive except in relation to stress symptoms (higher scores in the scale representing less stress symptoms) where the relationship was negative. For quality of care A, results of opportunities were non-significant. Support showed a positive relation to stress, job satisfaction, thriving and quality of care A. Resources showed positive relationships to stress, job satisfaction and thriving but no significant relationship to quality of care A or B. Informal power showed significant positive relationships to thriving and quality of care A and B. When adding leadership and management in the second model, Model 2, the relationships remained, except resources that no longer showed a statistically significant relationship to thriving at work. Management was revealed as being positively, statistically significantly related to stress symptoms, and leadership statistically significantly, negatively related to quality of care A and B. In the final model, Model 3, when controlling for covariates, the results for all outcomes remained except for the outcome variable quality of care B. For this outcome, additional factors from structural empowerment showed statistically significant relationships; support and formal power being negatively related while resources were positively related to quality of care B. Additionally, leadership no longer showed a statistically significant relationship to quality of care B.

**Table 4:** GEE models of relationships between empowering structures, management, leadership and wellbeing (individual level)

Individual level	Stress symptoms^3^					Job satisfaction^4^					Thriving at work^5^				
	Unstandardized coefficients B	95% Confidence interval			*P*-value	Unstandardized coefficients B	95% Confidence interval			*P*-value	Unstandardized coefficients B	95% Confidence interval			*P*-value
*Model 1*															
Opportunities^1^	**−3.874**	−6.625	to	−1.123	**0.006**	**0.166**	0.051	to	0.281	**0.005**	**0.348**	0.170	to	0.525	**< 0.001**
Information^1^	1.940	−0.435	to	4.315	0.109	−0.049	−0.132	to	0.034	0.250	−0.040	−0.132	to	0.052	0.396
Support^1^	**3.828**	0.766	to	6.890	**0.014**	**0.169**	0.061	to	0.276	**0.002**	**0.186**	0.018	to	0.354	**0.030**
Resources^1^	**8.050**	5.898	to	10.202	**< 0.001**	**0.119**	0.027	to	0.212	**0.011**	**0.127**	0.004	to	0.251	**0.043**
Formal power^1^	1.493	−1.565	to	4.550	0.339	0.069	−0.024	to	0.163	0.147	0.073	−0.068	to	0.214	0.312
Informal power^1^	1.665	−1.925	to	5.254	0.363	0.069	−0.068	to	0.205	0.324	**0.259**	0.101	to	0.417	**0.001**
*Model 2*															
Opportunities	**−3.534**	−6.047	to	−1.020	**0.006**	**0.174**	0.061	to	0.288	**0.003**	**0.355**	0.182	to	0.528	**< 0.001**
Information	1.424	−1.049	to	3.897	0.259	−0.062	−0.147	to	0.023	0.154	−0.051	−0.141	to	0.039	0.270
Support	**3.335**	0.594	to	6.076	**0.017**	**0.152**	0.046	to	0.259	**0.005**	**0.182**	0.002	to	0.361	**0.047**
Resources	**7.748**	5.712	to	9.785	**< 0.001**	**0.111**	0.014	to	0.207	**0.025**	0.125	−0.004	to	0.253	0.058
Formal power	1.205	−1.798	to	4.209	0.431	0.059	−0.034	to	0.151	0.213	0.074	−0.065	to	0.213	0.299
Informal power	1.676	−1.748	to	5.099	0.337	0.066	−0.064	to	0.195	0.319	**0.259**	0.110	to	0.407	**< 0.001**
Management^2^	**0.251**	0.082	to	0.419	**0.004**	0.005	0.000	to	0.010	0.062	0.007	0.000	to	0.014	0.063
Leadership^2^	−0.086	−0.241	to	0.068	0.274	0.000	−0.006	to	0.005	0.879	−0.004	−0.011	to	0.003	0.242
*Model 3*															
Opportunities	**−2.564**	−5.090	to	−0.038	**0.047**	**0.166**	0.037	to	0.295	**0.011**	**0.303**	0.121	to	0.486	**0.001**
Information	0.727	−1.906	to	3.361	0.588	−0.081	−0.172	to	0.009	0.078	−0.054	−0.156	to	0.048	0.299
Support	**2.931**	0.296	to	5.566	**0.029**	**0.141**	0.033	to	0.248	**0.011**	**0.198**	0.019	to	0.377	**0.030**
Resources	**7.659**	5.847	to	9.472	**< 0.001**	**0.113**	0.017	to	0.210	**0.021**	0.107	−0.033	to	0.247	0.135
Formal power	0.774	−2.164	to	3.711	0.606	0.046	−0.045	to	0.138	0.322	0.082	−0.060	to	0.225	0.257
Informal power	1.879	−1.607	to	5.365	0.291	0.063	−0.058	to	0.185	0.308	**0.231**	0.080	to	0.382	**0.003**
Management	**0.284**	0.130	to	0.438	**< 0.001**	0.004	−0.001	to	0.010	0.092	0.005	−0.002	to	0.013	0.158
Leadership	−0.099	−0.247	to	0.049	0.191	0.001	−0.006	to	0.007	0.848	−0.002	−0.009	to	0.005	0.567
Age	0.205	−0.020	to	0.429	0.075	**0.006**	0.000	to	0.012	**0.039**					
Gender						0.238	−0.061	to	0.537	0.119	**0.637**	0.219	to	1.055	**0.003**
Years in profession	−0.001	−0.018	to	0.017	0.946										
Years at unit											0.000	−0.001	to	0.001	0.467

*GEE* general estimating equations. Statistically significant values are marked with bold text. An empty cell means that the variable was not included in the analysis

^1^The conditions of Work Effectiveness Questionnaire; scale 1–5. ^2^The Leadership and Management Inventory; scale20–100. ^3^Satisfaction with Work Questionnaires; scale 0–100, higher values represent less stress in the present study.^4^Brief Index of Affective Job Satisfaction questionnaire; scale 1–5.^5^Thriving at work; scale 1–7

**Table 5:** GEE models of relationships between empowering structures, management, leadership and quality of care (individual level)

Individual level	Quality of care ICS-A^3^							Quality of care ICS-B^3^				
	Unstandardized coefficients B		95% Confidence interval				*P*-value	Unstandardized coefficients B	95% Confidence interval			*P*-value
*Model 1*												
Opportunities^1^	0.079	−0.021		to	0.179	0.121		**0.126**	0.021	to	0.232	**0.019**
Information^1^	−0.006	−0.079		to	0.067	0.867		0.001	−0.070	to	0.072	0.988
Support^1^	**0.074**	0.000		to	0.149	**0.049**		−0.024	−0.086	to	0.039	0.460
Resources^1^	0.043	−0.044		to	0.130	0.336		0.053	−0.038	to	0.143	0.252
Formal power^1^	−0.019	−0.102		to	0.064	0.659		−0.002	−0.086	to	0.082	0.957
Informal power^1^	**0.140**	0.059		to	0.221	**< 0.001**		**0.191**	0.108	to	0.274	**< 0.001**
*Model 2*												
Opportunities	0.081	−0.024		to	0.186	0.129		**0.130**	0.022	to	0.237	**0.018**
Information	0.000	−0.073		to	0.072	0.991		−0.001	−0.076	to	0.074	0.977
Support	**0.097**	0.021		to	0.173	**0.012**		−0.019	−0.085	to	0.047	0.580
Resources	0.045	−0.035		to	0.125	0.272		0.053	−0.037	to	0.144	0.249
Formal power	0.006	−0.082		to	0.093	0.899		0.008	−0.079	to	0.095	0.863
Informal power	**0.149**	0.072		to	0.227	**< 0.001**		**0.195**	0.115	to	0.275	**< 0.001**
Management^2^	0.003	−0.001		to	0.007	0.100		0.004	0.000	to	0.008	0.068
Leadership^2^	**−0.007**	−0.011		to	−0.003	**< 0.001**		**−0.004**	−0.008	to	−4.428E-5^b^	**0.048**
*Model 3*												
Opportunities	0.075	−0.025		to	0.174	0.140		**0.110**	0.048	to	0.173	**< 0.001**
Information	6.385E-5^a^	−0.072		to	0.072	0.999		0.040	−0.020	to	0.099	0.194
Support	**0.095**	0.020		to	0.169	**0.013**		**−0.104**	−0.173	to	−0.036	**0.003**
Resources	0.047	−0.033		to	0.127	0.252		**0.251**	0.185	to	0.317	**< 0.001**
Formal power	0.004	−0.084		to	0.093	0.921		**−0.124**	−0.202	to	−0.046	**0.002**
Informal power	**0.147**	0.068		to	0.226	**< 0.001**		**0.303**	0.227	to	0.379	**< 0.001**
Management	0.003	−0.001		to	0.007	0.172		−0.003	−0.007	to	0.001	0.191
Leadership	**−0.007**	−0.010		to	−0.003	**< 0.001**		0.000	−0.003	to	0.003	0.813
Age												
Gender	0.127	−0.134		to	0.388	0.341		0.229	−0.086	to	0.543	0.154
Years in profession												
Years at unit												

*GEE* general estimating equations. Statistically significant values are marked with bold text. An empty cell means that the variable was not included in the analysis

^a^6.385E-5, means 6.385 times ten to the minus five power, or 0.00006385 ^b^−4.428E-5, means −4.428 times ten to the minus five power, or −0.00004428

^1^The conditions of Work Effectiveness Questionnaire; scale 1–5

^2^The Leadership and Management Inventory; scale20–100

^3^Individualized care scale - nurse ICS-A-nurse; scale 1–5 and Individualized care scale - nurse ICS-B-nurse; scale 1–5

### Unit level

In Model 1 the factors support and resources showed to be positively statistically significantly related to the outcome variable stress symptoms (Table 6). Support also showed to be positively statistically significantly related to job satisfaction and thriving in Model 1. The factors opportunities and formal power was revealed as positively statistically significantly related to job satisfaction and the factor opportunities also showed the same relationship to thriving. The factor information showed a statistically significant, negative relationship to job satisfaction. None of the factors of structural empowerment showed any statistically significant relationships to quality of care in the first model (Table 7). In Model 2 when adding leadership and management, leadership showed a statistically significant, negative relationship to quality of care A and B. The other relationships, between the factors of structural empowerment and well-being showed in Model 1, remained, except for support no longer showing a positively statistically significant relationship to thriving. In Model 3, the results from Model 2 stayed the same.

**Table 6:** GEE models of relationships between empowering structures, management, leadership and wellbeing (unit level)

Unit level	Stress symptoms^3^					Job satisfaction^4^					Thriving at work^5^				
	Unstandardized coefficients B	95% Confidence interval			*P*-value	Unstandardized coefficients B	95% Confidence interval			*P*-value	Unstandardized coefficients B	95% Confidence interval			*P*-value
*Model 1*															
Opportunities^1^	−4.241	−10.974	to	2.492	0.217	**0.295**	0.080	to	0.510	**0.007**	**0.627**	0.124	to	1.130	**0.015**
Information^1^	−3.055	−6.768	to	0.658	0.107	**−0.171**	−0.323	to	−0.019	**0.028**	−0.207	−0.573	to	0.160	0.269
Support^1^	**9.387**	2.799	to	15.974	**0.005**	**0.412**	0.131	to	0.692	**0.004**	**0.967**	−0.001	to	1.935	**0.050**
Resources^1^	**12.187**	8.670	to	15.703	**< 0.001**	0.122	−0.041	to	0.285	0.144	0.038	−0.331	to	0.407	0.839
Formal power^1^	3.307	−1.486	to	8.101	0.176	**0.235**	0.100	to	0.371	**< 0.001**	0.042	−0.374	to	0.458	0.842
Informal power^1^	0.388	−6.468	to	7.245	0.912	−0.083	−0.336	to	0.170	0.522	−0.135	−0.545	to	0.275	0.518
*Model 2*															
Opportunities	−4.229	−10.942	to	2.483	0.217	**0.302**	0.098	to	0.506	**0.004**	**0.651**	0.172	to	1.130	**0.008**
Information	−2.863	−6.459	to	0.733	0.119	**−0.166**	−0.301	to	−0.031	**0.016**	−0.191	−0.520	to	0.137	0.254
Support	**9.398**	2.846	to	15.950	**0.005**	**0.398**	0.099	to	0.697	**0.009**	0.919	−0.053	to	1.891	0.064
Resources	**12.725**	7.818	to	17.633	**< 0.001**	0.116	−0.055	to	0.287	0.182	0.017	−0.346	to	0.379	0.927
Formal power	3.724	−1.279	to	8.728	0.145	**0.227**	0.083	to	0.372	**0.002**	0.013	−0.400	to	0.426	0.952
Informal power	0.781	−6.899	to	8.462	0.842	−0.061	−0.333	to	0.211	0.659	−0.064	−0.499	to	0.372	0.775
Management^2^	0.052	−0.221	to	0.325	0.709	0.003	−0.007	to	0.013	0.559	0.010	−0.005	to	0.025	0.190
Leadership^2^	−0.117	−0.317	to	0.082	0.249	−0.002	−0.010	to	0.006	0.601	−0.007	−0.021	to	0.007	0.358
*Model 3*															
Opportunities	−4.229	−10.942	to	2.483	0.217	**0.302**	0.098	to	0.506	**0.004**	**0.636**	0.154	to	1.118	**0.010**
Information	−2.863	−6.459	to	0.733	0.119	**−0.166**	−0.301	to	−0.031	**0.016**	−0.181	−0.511	to	0.150	0.283
Support	**9.398**	2.846	to	15.950	**0.005**	**0.398**	0.099	to	0.697	**0.009**	0.893	−0.031	to	1.816	0.058
Resources	**12.725**	7.818	to	17.633	**< 0.001**	0.116	−0.055	to	0.287	0.182	0.019	−0.337	to	0.375	0.916
Formal power	3.724	−1.279	to	8.728	0.145	**0.227**	0.083	to	0.372	**0.002**	0.034	−0.370	to	0.439	0.868
Informal power	0.781	−6.899	to	8.462	0.842	−0.061	−0.333	to	0.211	0.659	−0.057	−0.482	to	0.368	0.793
Management	0.052	−0.221	to	0.325	0.709	0.003	−0.007	to	0.013	0.559	0.009	−0.006	to	0.024	0.221
Leadership	−0.117	−0.317	to	0.082	0.249	−0.002	−0.010	to	0.006	0.601	−0.006	−0.020	to	0.007	0.349
Age															
Gender											**0.165**	0.002	to	0.329	**0.047**
Years in profession															
Years at unit											0.000	−0.001	to	0.000	0.184

*GEE* general estimating equations. Statistically significant values are marked with bold text. An empty cell means that the variable was not included in the analysis

^1^The conditions of Work Effectiveness Questionnaire; scale 1–5. ^2^The Leadership and Management Inventory; scale20–100. ^3^Satisfaction with Work Questionnaires; scale 0–100, higher values represent less stress in the present study. ^4^Brief Index of Affective Job Satisfaction questionnaire; scale 1–5.^5^Thriving at work; scale 1–7

**Table 7:** GEE models of relationships between empowering structures, management, leadership and quality of care (unit level)

Unit level	Quality of care ICS-A^3^						Quality of care ICS-B^3^					
	Unstandardized coefficients B		95% Confidence interval			*P*-value	Unstandardized coefficients B		95% Confidence interval			*P*-value
*Model 1*												
Opportunities^1^	0.182	−0.121		to	0.485	0.240	0.137	−0.107		to	0.381	0.271
Information^1^	0.013	−0.178		to	0.205	0.891	0.065	−0.079		to	0.210	0.376
Support^1^	0.183	−0.191		to	0.558	0.338	−0.040	−0.283		to	0.202	0.745
Resources^1^	−0.014	−0.225		to	0.196	0.894	−0.048	−0.223		to	0.127	0.592
Formal power^1^	0.027	−0.225		to	0.279	0.835	0.179	−0.045		to	0.403	0.118
Informal power^1^	−0.033	−0.320		to	0.254	0.821	0.160	−0.037		to	0.358	0.111
*Model 2*												
Opportunities	0.160	−0.103		to	0.424	0.234	0.140	−0.084		to	0.364	0.221
Information	0.030	−0.123		to	0.184	0.701	0.080	−0.042		to	0.202	0.197
Support	0.233	−0.007		to	0.474	0.057	−0.044	−0.233		to	0.145	0.649
Resources	0.097	−0.105		to	0.298	0.348	−0.012	−0.218		to	0.194	0.910
Formal power	0.126	−0.101		to	0.353	0.277	0.205	−0.014		to	0.425	0.067
Informal power	−0.039	−0.279		to	0.202	0.751	0.195	−0.015		to	0.405	0.069
Management^2^	−0.001	−0.014		to	0.011	0.848	0.005	−0.010		to	0.019	0.548
Leadership^2^	**−0.013**	−0.020		to	−0.006	**< 0.001**	**−0.009**	−0.014		to	−0.004	**< 0.001**
*Model 3*												
Opportunities	0.169	−0.093		to	0.431	0.206	0.140	−0.084		to	0.364	0.221
Information	0.028	−0.123		to	0.179	0.715	0.080	−0.042		to	0.202	0.197
Support	0.221	−0.012		to	0.454	0.063	−0.044	−0.233		to	0.145	0.649
Resources	0.099	−0.101		to	0.299	0.333	−0.012	−0.218		to	0.194	0.910
Formal power	0.119	−0.100		to	0.339	0.286	0.205	−0.014		to	0.425	0.067
Informal power	−0.030	−0.265		to	0.206	0.805	0.195	−0.015		to	0.405	0.069
Management	−0.001	−0.013		to	0.012	0.891	0.005	−0.010		to	0.019	0.548
Leadership	**−0.013**	−0.020		to	−0.006	**< 0.001**	**−0.009**	−0.014		to	−0.004	**< 0.001**
Age												
Gender												
Years in profession												
Years at unit	0.000	−3.899E5^a^		to	0.000	0.122						

*GEE* general estimating equations. Statistically significant values are marked with bold text. An empty cell means that the variable was not included in the analysis

^a^3.899E-5, means −3.899 times ten to the minus five power, or −0.00003899

^1^The conditions of Work Effectiveness Questionnaire; scale 1–5. ^2^The Leadership and Management Inventory; scale20–100

^3^Individualized care scale - nurse ICS-A-nurse; scale 1–5 and Individualized care scale - nurse ICS-B-nurse; scale 1–5

## Discussion

From the main findings in this study, the empowering structures that consisted of opportunities, support and informal power could be seen as factors of importance for both the individual and the unit in relation to staff well-being and the quality of care. Additionally, resources showed to be significantly positive to quality of care and job satisfaction at the individual level and to stress symptoms at both the individual and unit level. These main findings regarding structural empowerment are all in line with previous research [14, 24–26, 28, 29] and therefore expected. Results that were not expected, but are of interest, are the non-significant results for leadership and management performance in relation to staff well-being and quality of care. When adding leadership and management in our GEE-models, there were a lack of relationships towards well-being and quality of care, which stands in contrast to previous research findings [3, 34, 37]. However, it is important to remember that according to theory and previous research, employees perceived access to empowering structures most likely is influenced by their manager’s perceived access to structural empowerment [7, 18]. This influence can occur over time [7] and since our results derives from cross sectional data, the significance of the current FLMs’ leadership and management performance could be concealed by earlier possible work by current and/or former managers along with foundations made by the organizations to make empowering structures accessible to their staff. Conducting future longitudinal studies could help to track changes over time and allow to examine causal relationships and long-term effects. However, the hospital settings evolving nature presents challenges for consistent data collection over time [51]. Further, it could be argued that FLMs who have established access to empowering structures for their staff has effectively fulfilled their managerial responsibilities, which may explain why the LaMI did not yield the expected results. The LaMI-instrument includes items reflecting behaviors of transformational leadership [46], and links between transformational leadership and structural empowerment among staff have been shown in earlier research [52], suggesting that effective leadership can enhance nurses’ perceptions of access to structural empowerment. An alternative approach to measuring the influence of FLMs managerial practice is to assess a less ideal leadership style instead (c.f [36]). Moreover, the non-significant results for leadership and management performance in relation to quality of care in the GEE models could perhaps be explained by how nursing staff strive to deliver the best possible care regardless of the features of their work environment or their leaders’ actions, demonstrating dedication and resilience even under challenging circumstances [53].

Our results showed that empowering structures are linked to staff well-being, as confirmed by several previous studies [10, 24, 26]. Such results ought to motivate hospital management to promote access to empowering structures. At our individual level of analysis, access to empowering structures were related to quality of care, as shown in the very early work by Laschinger and colleagues [54]. That informal power positively relates to the individual’s assessed quality of care in our results, can also be supported by Laschinger’s early research, which states that nurses with strong alliances in the organization also rate higher levels of control in their nursing practice [11]. However, support and formal power showed negative relationships towards individual ratings of care quality perceived during the last work shift. These results can perhaps be understood by the individual’s own perception that they were not fully able to live up to the responsibilities that are fundamental in their role. Surprisingly, at the unit level, none of our independent variables in the multivariate analysis showed any significant relationships regarding quality of care, except for leadership that showed a negative relationship in the lower end of significance. These results call for further investigation, preferably with a larger sample size to better capture any potential outcomes at the unit level. Using alternative outcome measures could illuminate patients’ experiences of the care process and their perceptions of the quality of care provided (c.f [55]). Additionally, outcome measures of patient safety, like reports of adverse events, could be an alternative for measuring the outcome of the care process [10]. Another not expected result at the individual level, was the negative relationship between access to opportunities and stress symptoms, which in our study indicates that greater access to opportunities is related to individuals perceiving stress symptoms more often. In line with our expectations, positive relationships for the factor opportunities were revealed regarding our other well-being variables. However, a reasonable explanation is that individuals with additional access to opportunities, when the other factors in our analysis are held constant, could respond with stress since it is adding possibilities to learn and grow without additional access to other empowering structures. Access to information, at unit level, also showed a negative relationship towards well-being in terms of job satisfaction. According to Kanter’s theory [18], access to information is important for staff well-being and work performance and findings in previous research using the CWEQ-instrument has confirmed that. However, lower job satisfaction has been listed as a symptom of information overload in an interdisciplinary review [56]. And within nursing research, calls for developing strategies with the aim to process the excessive information received by nursing staff on a daily basis are arising [57, 58].

The strained work situation within hospitals today, emphasizes the need to understand and form strategies to address the situation. Similar to the focus on structural empowerment within nursing research, the concept of resilience is often discussed as a key to manage the constant challenges and strains nursing staff are facing on a daily basis [59, 60]. To achieve organizational resilience, the factors described to be of importance are; resource availability, supportive and learning oriented cultures, shared power and responsibility [61]. This is in line with the empowering structures described in Kanter’s theory and the focus of our research. However, within nursing research, the focus is often on the resilience of the individual nurse. There is criticism regarding interventions made for strengthening and building the resilience of the individual nurse, since the focus is on individual and internal factors rather than the unit or organizational factors [62]. With the aim of creating better working conditions, it is, as shown in our results, important to distinguish between how empowering structures relate regarding the individual and unit levels, and how they relate to staff well-being and quality of care. Our results emphasize the need to differentiate how empowering structures and managerial practices relate at both the individual and unit levels and their relations to staff well-being and quality of care. Some factors, such as support and resources, relates to both individual and unit outcomes. In contrast, others, like management, leadership and informal power, primarily relate to individuals without translating to the unit-level. This distinction could be important for designing effective interventions. If the goal is to enhance both individual and unit outcomes, interventions must target factors that influence both levels. Conversely, if the focus is solely on individual wellbeing, attention should be directed to factors that specifically impact personal experiences. By a deeper understanding of these relationships at both the individual and unit levels, healthcare organizations could tailor interventions to achieve desired outcomes in staff wellbeing and quality of care.

### Strengths and limitations

The greatest strength of this study was its sampling method, which resulted in a representative sample of the medical and surgical nursing staff at Swedish hospitals. However, the findings should be interpreted with some limitations in mind. First, the cross-sectional design with self-reported data hinders any conclusions regarding causality. Second, is the potential risk for common method variance when self-reported data are gathered and analyzed. However, the instruments used could be seen as established and validated, and the data analyses chosen indicated a low risk for response biases. That the aggregated unit level data are based on responses from 2 to 14 individuals at each unit could be seen as a weakness, but only one unit had data based on two individuals due to internal missing data. The majority of the units included in the GEE-analyses had data based on responses from 9 to 13 individuals.

## Conclusions

The results confirm the importance of staff access to empowering structures in relation to staff well-being and the quality of care. Additionally, differences and similarities were shown when studying these relationships at the individual and unit level. These results motivate further research regarding different levels of analysis and if possible, with longitudinal design. The results also imply further research studying the performances and structures of organizations, in favor of a traditional focus on the individual performances of managers within the field of leadership. Further, results from this study could be used as guidance when planning an intervention or implementing strategies with the aim to promote staff well-being and quality of care. It is important to focus on the intended level, individual vs. unit, when implementing access to empowering structures. Management has a responsibility and an obligation to establish empowering organizational structures. At the same time, when management encourage and offer ways of development and learning, our results imply that an inventory of staff access to additional empowering structures could be of importance for achieving positive outcomes in terms of staff wellbeing.

## Data Availability

The data supporting the findings of this study are available from the corresponding author upon reasonable request.

## Abbreviations

GEE: General Estimation Equation
FLM: First-Line Manager
ICS: Individualized Care Scale
STROBE: Strengthening The Reporting of Observational studies in Epidemiology
CWEQ-II: Conditions for Work Effectiveness Questionnaire
BIAJS: Brief Index of Affective Job Satisfaction
